# From conformal infinity to equations of motion: conserved quantities in general relativity

**DOI:** 10.1098/rsta.2023.0041

**Published:** 2024-03-04

**Authors:** Roger Penrose

**Affiliations:** Mathematical Institute, Oxford, Oxfordshire OX2 6GG, UK

**Keywords:** general relativity, conformal infinity, equations of motion

## Abstract

This paper describes conservation laws in general relativity (GR) dating back to the mass-energy conservation of Bondi and Sachs in the early 1960s but using 2-spinor techniques. The notion of conformal infinity is employed, and the highly original ideas of E. T. Newman are discussed in relation to twistor theory. The controversial NP constants are introduced, and their meaning is considered in a new light related to the problem of equations of motion in GR.

This article is part of a discussion meeting issue ‘At the interface of asymptotics, conformal methods and analysis in general relativity’.

## 1. Early work on gravitational radiation

The late 1950s and early 1960s saw the beginnings of our current understanding of gravitational radiation, according to general relativity (GR), and how gravitational waves carry energy away from a system of accelerating gravitating bodies. Already in 1925 [1], H. W. Brinkmann had discovered the metric for a gravitational plane wave (subsequently commonly referred to as a *pp*-wave), but his solution was not at all well known to the physicists at that time. Later considerations of gravitational plane waves, using what had seemed to be a more directly appropriate type of coordinate system, led to much confusion, as it had seemed that singularities would necessarily occur in these solutions when extended too far into the future or past. Einstein himself, in his 1937 collaboration with Rosen [2], had seemed to conclude that plane gravitational waves did not properly exist in GR, and they re-interpreted their solution as describing *cylindrical* waves instead! This confusion was later greatly clarified, by Ivor Robinson, as expressed in his 1959 paper [3] with Hermann Bondi and Felix Pirani, using a coordinate patchwork. The basic underlying conundrum was later identified, in 1965, in the curious fact that a gravitational plane wave does not admit a global *Cauchy hypersurface* (i.e. a spacelike three-surface that intersects every maximally extended time-like curve—or path—in the space–time). (See [4]).

Although it is a normal procedure in other areas of physics to decompose the radiation field into plane-wave components, the considerations raised above suggest that such a procedure might well encounter serious difficulties in GR. Moreover, the very nonlinear nature of GR presents additional difficulties for such a procedure, and very different techniques turned out to be much more effective for providing an understanding of gravitational radiation in accordance with GR.

Another concern of historical importance was the issue of whether massive bodies moving about one another solely under their mutual gravitational influences would, according to GR, actually radiate gravitationally, or even respond to the presence of a gravitational-wave radiation. Back in 1918 [5], Einstein had shown that in the linear weak-field limit of GR, a change in the mass quadrupole moment of a massive system would result in the emission of gravitational waves. However, not only was this a result directly applicable only to this linear approximation of GR, but it also did not supply an answer to how such radiation might carry away energy or influence the motions of other bodies. More importantly, non-gravitational forces might be needed in order to effect the required changes in quadrupole moment of the masses involved, so this result, albeit an important one, did not directly answer the question of whether gravitational waves might arise from purely gravitationally caused motions.

Accordingly, this left open the issue, raised particularly by the Polish physicist Leopold Infeld, of whether gravitational waves would indeed be emitted if the sources were accelerating only under the influence of their own mutual gravitational interactions. Einstein's famous 1938 paper, with Infeld and Hoffmann [6], addressed the issue of how a system of gravitating bodies, considered to be point-like in some appropriate limit, would move under their mutual gravitational attractions constrained only by the GR field equations, thus going beyond Einstein's GR *assumption* of geodetic motion for point-like test-particles. However, this EIH paper did not seem to give a clear overall answer to these questions. Indeed, Infeld had been of the strong opinion that bodies, interacting with each other entirely gravitationally, would not actually emit gravitational waves.

It should be pointed out that there are some serious issues concerning what is specifically meant by a gravitating ‘point particle' in GR, since for a ‘positive mass point particle', we would actually have to consider a tiny black hole (or perhaps a ‘white hole’ or even possibly both at once!) rather than a point source. This is the GR version of a general issue that arises with equations of motion. In electromagnetism, for example, the motion of a charged particle in an ambient electromagnetic field should be influenced only by the *background* field in which it moves, whereas the *actual* field, very close to the particle itself, would be dominated by the particle's own field, rather than the background field that it is supposed to be responding to. Issues of this nature will be a main concern of the later considerations of this article.

The fact that gravitational waves could actually be emitted by purely gravitating systems, despite Infeld's frequently expressed opinion, was strongly indicated by the work of Trautman in [7], (partly influenced by Jerzy Plebański). Later, Trautman joined forces with Ivor Robinson to obtain a beautiful special family of radiating vacuum solutions with gravitational waves coming radially outwards from a localized source [8,9].

However, it was Hermann Bondi's work on axi-symmetric spacetimes, initially presented in outline, in *Nature* in1960 [10], but then worked out in considerable detail, with his students van der Burg and Matzner, published in 1962) [11], that greatly clarified the picture. In particular, this paper identified a specific quantity *M*, which could be identified as the *total retarded mass-energy* of the system—where the notion or ‘retarded', as used here, will be explained shortly; see (1.1). Importantly, the value of *M* was shown to be reduced, as the (retarded) time parameter *u* progresses. Thus, we can take the view that part of *M* is ‘carried away' by the outgoing gravitational radiation, by an amount that can be identified in a certain *positive* term in the outgoing radiation field.

Soon afterwards the restriction of axi-symmetry was importantly removed by Sachs [12], this work providing a clear demonstration that (positive) energy-carrying gravitational waves do indeed occur in GR, in precise accordance with Einstein's original expectations arising from his early considerations with the linear limit of GR [5]. In particular, there was provided a clear mass-energy conservation law, showing that the sum of the retarded mass-energy of the gravitating sources, together with the mass-energy carried away by the outgoing radiation, would remain constant throughout the entire process. Moreover, the contribution from the gravitational radiation, whenever present at all, would be necessarily *positive*, resulting in a reduction in the total (retarded) mass-energy *M* of the system whenever outgoing radiation is present.

The Bondi-Sachs approach was based on a form of coordinate system in which one of the coordinates, *u*, regarded as a *retarded time* parameter, the constant values of which, at least sufficiently far from the sources, would describe topologically spherical surfaces moving outwards with the speed of light, so that the *u *= constant hypersurfaces would be *null*,

1.1 $$g^{ab}\;\nabla_{a}u\nabla_{b}u=0,$$

with null-geodesic generator lines that extend outwards to a future null infinity, as they move away from the source region. In addition to the coordinate *u*, there would be an outward radial coordinate *r*, that could be taken to be an *affine parameter* along each null-geodesic generator (although Bondi actually chose what he referred to as a *luminosity parameter* that differed slightly from an affine parameter, this difference being unimportant for our considerations here, and plays no role in the discussions that follow).

The two remaining coordinates can be taken to be standard spherical polar *θ* and *ϕ*, which are taken to remain unchanged along the null-geodesic generators, and provide the standard two-spherical form

1.2 $$r^{2}(d\theta^{2}+\text{si}{\text{n}}^{2}\theta \;\text{d}\phi^{2}),$$

in the limit of large *r*, this imposing a restriction on how the affine parameter *r* is chosen on the various generators of the outgoing null hypersurfaces *u *= const., a matter that will be clarified in more detail in §3. This work was an especially important development, because one could identify a quantity *M* that could be interpreted as the *mass*(-energy) of the system at each retarded time (*u* = const.) and that this mass measure *M*, now regarded as a function *M*(*u*) of the retarded time *u* so that *M*(*u*) is necessarily a never-increasing function of *u*, which was a very satisfactory result, from the physical point of view, as it assigned a clear-cut positive value to the mass-energy carried away by gravitational radiation. The actual positivity of *M* itself was a separate matter, and results of importance here were achieved by Schoen & Yau [13–15], Witten [15], Ruela & Tod [16], Horowitz & Perry [17], Ludvigsen & Vickers [18,19]).

Nevertheless, at the time when Bondi first produced his important contributions to this work, he was still not completely free of Infeld's influence, making the curious tentative suggestion that certain apparently ‘non-radiative but non-static motions' that appear in his considerations might possibly represent Infeld's supposed radiation-free motions under gravity. However, the formalism was not really set up in a way that might eliminate *incoming* gravitational radiation despite the expressed hope, in the paper, that such incoming waves had been excluded (see also [20]). Indeed, we shall be seeing in §3 that the ‘retarded’ (*u*, *r*, *θ*, *ϕ*) coordinate system is not at all suitable for formulating a condition expressing the absence of incoming radiation, and we may take it that Bondi's supposed ‘non-radiative but non-static motions' must actually have involved the presence of *incoming* gravitational radiation. A broader perspective on this issue will be provided in §3.

## 2. The 2-spinor formalism

In order to express concisely the quantities occurring in the following sections, it will be convenient to incorporate the 2-spinor abstract-index formalism. Details of this, and the relevant expressions, can be found in *Spinors and Space-Time* [21,22], but a very brief outline will be indicated here. In conventional notation, an expression such as

2.1 $${T_{c}}^{ab},$$

for example, would denote the array of components, in some coordinate system or local basis frame, of a tensor quantity ***T*** where *a, b*, *c* range over some set of alternative values, say 1, 2, 3, … , *n.* In the *abstract-index notation*, on the other hand, the expression (2.1) would instead denote the entire tensor ***T*** itself, but together with certain elements *a*, *b*, *c*, taken from the tensor index labelling set

2.2 $$S=\{a,b,c,d,\;\ldots ,z,a_{0},b_{0},\;\ldots a_{1},\;\ldots \},$$

where no coordinate system or local reference frame is involved. If, however, we do wish to refer to the family of components of ***T*** with respect to some local reference system, then the notation

2.3 $${T_{\text{c}}}^{\text{ab}},$$

with bold-face upright indices, would be used (where also hybrid expressions using both types of index in the same expression are also allowed). Thus, for ordinary four-dimensional spacetime, whereas the expression (2.3) stands for an array of 64 numbers or functions, in the usual way, where the expression (2.1) stands for a single tensorial quantity.

The utility of the abstract-index notation is not immediately evident, but is made particularly manifest in the *2-spinor formalism*, where we can use *capital* italic abstract-index letters for the two-dimensional complex spin-space at each point and *primed* italic capital letters for the corresponding *complex-conjugate* spin space. A particular strength of the abstract-index notation is that it allows us to exploit the power of the 2-spinor formalism in a simple and manifest way without the need to clutter expressions with translation quantities such as Infeld-van der Waerden translation symbols (see [23,24]). This is achieved by regarding each abstract spacetime index as the composite (product) of an un-primed and a primed capitalized version of the chosen tensor-index letter

2.4 $$a=AA^{\prime },b=BB^{\prime },c=CC^{\prime },d=DD^{\prime },\ldots ,$$

so that our tensor ***T*** can be expressed in a spinor form as

2.5 $${T_{c}}^{ab}={T_{CC^{\prime }}}^{AA^{\prime }BB^{\prime }},$$

or in a hybrid form such as ${T_{c}}^{aBB^{\prime }}$ etc.

The 2-spinor formalism provides a lot of power and flexibility that is not easy to express in the conventional tensor formalism for spacetime. For example, the vector quantity

2.6 $$S^{a}={T_{CC^{\prime }}}^{CA^{\prime }AC^{\prime }},$$

is easily expressed in the 2-spinor formalism, whereas its far-from-obvious expression in terms of conventional tensor notation turns out to be remarkably complicated!

Since the primed and un-primed spin-spaces are algebraically different spaces (albeit complex conjugates of one another), we can, without ambiguity, commute a primed index with an un-primed one (as was implicit in the interchange of *A′* with *A* in (2.6)), although we must keep track of the ordering of the un-primed indices and of the primed ones separately. This is helpful for writing symmetric and skew-symmetric parts (using round brackets or square brackets, respectively). For example

2.7 $${T_{CC^{\prime }}}^{AA^{\prime }BB^{\prime }}={T_{CC^{\prime }}}^{A^{\prime }ABB^{\prime }},$$

so, we can express

2.8 $${T_{CC^{\prime }}}^{A^{\prime }(AB)B^{\prime }}=\frac{1}{2}({T_{CC^{\prime }}}^{A^{\prime }ABB^{\prime }}+{T_{CC^{\prime }}}^{A^{\prime }BAB^{\prime }}).$$

Thus, we do need to keep the ordering within the system of un-primed and also within the system of primed indices, though the ordering between the two is irrelevant. Moreover, in view of the fact that spinor indices will need to be allowed to be raised or lowered, consistently with that process as applied to tensor indices, we shall need to be able to keep track of this ordering, both for the un-primed and for the primed indices. Accordingly, we need to stagger the upper and lower indices so that the necessary ordering is preserved, with a clearly determined space for each index so that it is unambiguous what this ordering is intended to be, when raised or lowered.

**Figure 1.**
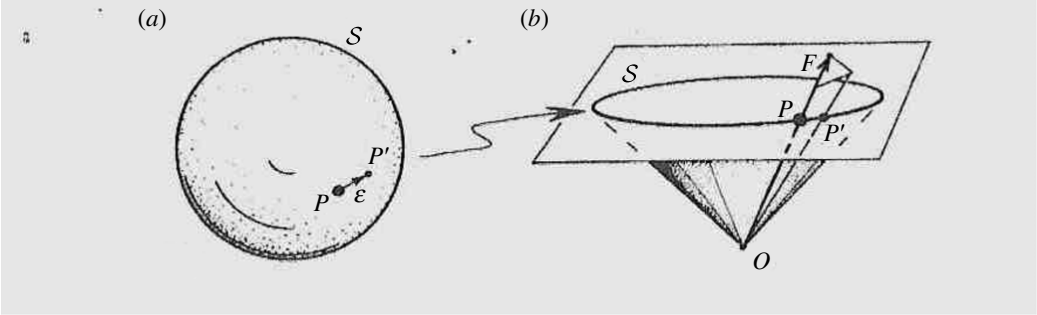
A spin-vector, pictured as a future-null vector ‘flagpole', determining a point on the future-celestial (Riemann) sphere with a null half-plane ‘flag' attached, to describe (up to sign) the spinor's phase.

Although the 2-spinor formalism has a considerable value in simplifying tensor expressions, where the primed and un-primed spinor indices are equal in number, it will be of importance for us that an individual *spin-vector* (i.e having a single spinor index) also has a geometrical interpretation (up to a sign). This is as a future-pointing null vector (its ‘flagpole') with a null half-plane attached to it (the ‘flag' attached to it to describes the spinor's *phase* [21] (figure 1).

The ratio of the two complex components of the spin-vector provides a point on the (future) celestial sphere, but the flag-plane fixes the phase, whose sign reverses under 2π rotation.

Raising or lowering spinor indices corresponds to the same process as for tensors, this requiring the symmetric metric tensor *g_ab_* for lowering tensor indices and its inverse *g^*ab*^* for raising them. In the case of spinors, we have *skew-symmetrical* spinors $\varepsilon_{AB}$ and $\varepsilon_{A^{\prime }B^{\prime }}$, for lowering spinor indices and $\varepsilon^{AB}$ and $\varepsilon^{A^{\prime }B^{\prime }}$ for raising them, where

2.9 $$g_{ab}=\varepsilon_{AB}\varepsilon_{A^{\prime }B^{\prime }},\;g^{ab}=\varepsilon^{AB}\varepsilon^{A^{\prime }B^{\prime }}.$$

Since these epsilon quantities are skew, we have to be careful about the ordering of the indices, and we write

2.10 $$X^{A}=\varepsilon^{AB}X_{B},\;X_{B}=X^{A}\varepsilon_{AB},\;Y^{A^{\prime }}=\varepsilon^{A^{\prime }B^{\prime }}Y_{B^{\prime }},\;Y_{B^{\prime }}=Y^{A^{\prime }}\varepsilon_{A^{\prime }B^{\prime }},$$

and we find (being careful about the index ordering) that that ${\varepsilon_{A}}^{B}=-{\varepsilon_{A}}^{B}$ and ${\varepsilon_{A^{\prime }}}^{B^{\prime }}=-{\varepsilon_{A^{\prime }}}^{B^{\prime }}$ are ‘Kronecker delta' identity symbols. It should also be noted that the epsilon spinors satisfy the identities

2.11 $$\begin{array}{c} \varepsilon_{AB}\varepsilon_{CD}+\varepsilon_{AC}\varepsilon_{DB}+\varepsilon_{AD}\varepsilon_{BC}=0,\\ \varepsilon_{A^{\prime }B^{\prime }}\varepsilon_{C^{\prime }D^{\prime }}+\varepsilon_{A^{\prime }C^{\prime }}\varepsilon_{D^{\prime }B^{\prime }}+\varepsilon_{A^{\prime }D^{\prime }}\varepsilon_{B^{\prime }C^{\prime }}=0, \end{array}\}$$

and the raised versions of these identities.

It is often convenient to introduce a reference *spin-frame* {*o*^*A*^, $\iota^{A}$} for the un-primed spin-space and {$o^{A^{\prime }}$, $\iota^{A^{\prime }}$} (usually written without the over-bars) for the complex conjugate spin-frame, for the primed spin-space. Accordingly, a typical component, in this spin-frame, of a spinor quantity $\varphi_{ADCD^{\prime }E^{\prime }}$ would be, for example,

2.12 $$\varphi_{1011^{\prime }0^{\prime }}=\varphi_{ABCD^{\prime }E^{\prime }}\iota^{A}o^{B}\iota^{C}\iota^{D^{\prime }}o^{E^{\prime }}.$$

It is normal to take a spin-frame to be normalized, so that

2.13 $$o_{A}\iota^{A}=1,\;o_{A^{\prime }}\iota^{A^{\prime }}=1,$$

from which it follows that the raising of a lower-index 1 or 1^′^ on a spinor quantity becomes a raised 0 or 0′, respectively, and the raising of a lower 0 or 0′ on a spinor quantity becomes a raised 1 or 1′, respectively, but with a change of sign for each such index.

A spin-frame is very closely related to a *null tetrad*, which consists of two future-pointing null vectors *l^*a*^* and *n^*a*^* and a complex null vector *m*^*a*^, which arise from a normalized spin frame according to

2.14 $$l^{a}=o^{A}o^{A^{\prime }},\;n^{a}=\iota^{A}\iota^{A^{\prime }},\;m^{a}=o^{A}\iota^{A^{\prime }},\;{\bar{m}}^{a}=\iota^{A}o^{A^{\prime }},$$

so that the only two non-zero scalar products between these vectors are

2.15 $$l_{a}n^{a}=1\;\text{and}\;m_{a}{\bar{m}}^{a}=-1.$$

In vacuum spacetime, with zero cosmological constant (Λ = 0), the Riemann curvature tensor *R_abcd_* is equal to the Weyl conformal tensor *C_abcd_*, where it turns out that, in spinor form [21,22,25]:

2.16*a* $$C_{abcd}=\Psi_{ABCD}\varepsilon_{A^{\prime }B^{\prime }}\varepsilon_{C^{\prime }D^{\prime }}+\varepsilon_{AB}\varepsilon_{CD}{\bar{\Psi }}_{A^{\prime }B^{\prime }C^{\prime }D^{\prime }},$$

so that the curvature can be completely described by the totally symmetric spinor

2.16*b* $$\Psi_{ABCD}=\Psi_{\left(ABCD\right)},$$

(see [25]). Moreover (as follows from the ‘fundamental theorem' that any complex polynomial in one variable is a product of linear factors), that we can express this as the symmetrized product of four single-indexed spinors

2.17 $$\Psi_{ABCD}=\alpha_{(A}\beta_{B}\gamma_{C}\delta_{D)},$$

each of which determines a null direction, as in figure 1 (via the null vectors$\alpha^{A}{\bar{\alpha }}^{A^{\prime }},\beta^{A}{\bar{\beta }}^{A^{\prime }},\text{etc}.)$, these four null directions being called the *principal null directions*, or *PND*, of the Weyl tensor (see [25]).

It provides a useful classification of particular solutions of the Einstein equations to take note of the coincidences between PND [21,22]. For example, the plane waves considered in §1 (pp-waves) are all type {4}, i.e. all PND coincide, this case being referred to as *null*, whereas the Schwarzschild and Kerr [26,27] solutions are all type {22} (sometimes called ‘type D'), i.e. with the PND coinciding in two separate pairs. It had been an early observation of Trautman [7] that the leading term (*r*^–1^-term) in a gravitationally radiating system would be *null*, i.e. type {4}.

**Figure 2.**
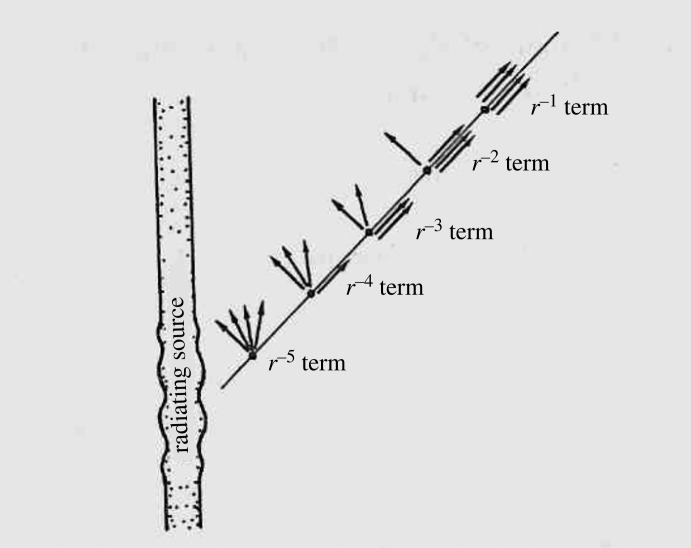
The Sachs peeling property.

Most revealing was the *peeling property* discovered by Sachs [12]. What Sachs found was that not only would the leading *r*^−1^ have all its PND pointing radially outwards (null case), as Trautman had already found, but the next *r*^−2^ term would have *three* PND pointing outwards, the *r*^−3^ term would have *two* PND pointing outwards, and the *r*^−4^ term would have *one* such PND. Thus, the picture is presented of the PND *peeling off*, one-by-one, as we move inward from infinity (figure 2)! It should, however, be pointed out that this is what would be expected, according to Sachs's analysis, in the *general* case. In special situations some of these terms might vanish, or perhaps just be more special, with more outward PND, this being understood, in the general picture presented by Sachs.

I first heard of this ‘peeling' behaviour when I was in Syracuse, New York state in the early few months of 1961. I had been struck by its remarkable elegance, but it took several months, until after I had returned to England, before I recognized its true geometrical significance. Yet, a month or so earlier than first hearing of Sachs's remarkable result, l had attended Trautman's talk, given in Syracuse, where he showed that the leading *r*^–1^ term in a gravitationally radiating system would indeed be *null*, i.e. type {4}. However, I was somewhat daunted by Trautman's rather complicated calculations, and I had wondered whether there might be a more geometrical way of understanding his result. I had toyed with the thought that perhaps one might simplify things by employing something like a conformal ‘inversion' to bring infinity to a finite location. However, I soon realized that even with the Schwarzschild spacetime, this would not work, because the inversion would exchange the origin point with a point at infinity, that being conformally *singula*r in the Schwarzschild case—as we would now understand as the singular point **i^0^** in the conformal picture. This was no help at all; so that at that time (early 1961, in Syracuse) I had given up on the ‘conformal inversion' idea.

However, some six months later, while back in the UK, I started to think again about Ray Sachs's very striking ‘peeling property'. I began to realize that if we move off in a *null* direction rather than a spacelike one, in the conformal picture, the limits are much more favourable (mainly because we need to balance only a relatively mild *r*^–1^ behaviour, rather than the more serious *r*^–2^ divergence that we encounter in spacelike directions), and I soon realized that Sachs's peeling behaviour is simply a statement about how the *different components* of the gravitational field, as defined appropriately from the Weyl spinor Ψ*_ABCD_*, behave at *null* infinity! This realization was the key to the utility of the conformal approach to the study of gravitational radiation, and its relation to the radiation of other massless fields in relativity theory. We examine the details of this in the next section.

## 3. At scri: $I^{+}$

Let us consider a general *conformal rescaling* of the metric, and other relevant quantities by a smoothly varying scalar quantity Ω, known as the *conformal factor*. When applied directly to the metric, we get

3.1 $$g_{ab}\mapsto {\tilde{g}}_{ab}=\Omega^{2}g_{ab}\;\text{and}\;g^{ab}\mapsto {\tilde{g}}^{ab}=\Omega^{-2}g^{ab},$$

which we accompany by

3.2 $$\begin{array}{lll} & \varepsilon_{AB}\mapsto {\tilde{\varepsilon }}_{AB}=\Omega \varepsilon_{AB}, & \varepsilon^{AB}\mapsto {\tilde{\varepsilon }}^{AB}=\Omega^{-1}\varepsilon^{AB}\\ \;\text{and}\; & \varepsilon_{A^{\prime }B^{\prime }}\mapsto {\tilde{\varepsilon }}_{A^{\prime }B^{\prime }}=\Omega \varepsilon_{A^{\prime }B^{\prime }}, & \varepsilon^{A^{\prime }B^{\prime }}\mapsto {\tilde{\varepsilon }}^{A^{\prime }B^{\prime }}=\Omega^{-1}\;\varepsilon^{A^{\prime }B^{\prime }}. \end{array}\}$$

The massless free-field equations for non-zero spin *s* can be written as

3.3 $$\nabla^{AA^{\prime }}\phi_{AB\ldots E}=0,$$

where *ϕ_AB_*_…_*_E_* (with 2 *s* indices, where *s*> 0 is an integer or half-integer), is totally symmetric

3.4 $$\phi_{AB\ldots E}=\phi_{\left(AB\ldots E\right)}.$$

Equation (3.3) is conformally invariant if *ϕ_AB_*_…_*_E_* scales as a density of weight –1 under the conformal re-scaling (3.1), (3.2)

3.5 $$\phi_{AB\ldots E}\mapsto {\tilde{\phi }}_{AB\ldots E}=\Omega^{-1}\;\phi_{AB\ldots E}.$$

This invariance follows from the way that the *covariant derivative* operator $\nabla_{a}$ changes under conformal rescaling. Defining

3.6 $$\Upsilon_{a}=\Omega^{-1}\nabla_{a}\Omega =\nabla_{a}\text{log}\Omega$$

we find that if *κ_A_* and $\mu_{A^{\prime }}$ are conformal densities of respective weights *w* and *w*^′^

3.7 $$\kappa_{A}\mapsto {\tilde{\kappa }}_{A}=\Omega^{w}\kappa_{A},\;\mu_{A^{\prime }}\mapsto {\tilde{\mu }}_{A^{\prime }}=\Omega^{w^{\prime }}\mu_{A^{\prime }},$$

then their covariant derivatives transform as

3.8 $$\begin{array}{ll} & \nabla_{AA^{\prime }}\kappa_{B}\mapsto {\tilde{\nabla }}_{AA^{\prime }}{\tilde{\kappa }}_{B}=\Omega^{w}(w\Upsilon_{AA^{\prime }}\kappa_{B}+\nabla_{AA^{\prime }}\kappa_{B}-\Upsilon_{BA^{\prime }}\kappa_{A})\\ \;\text{and}\; & \nabla_{AA^{\prime }}\;\mu_{B^{\prime }}\mapsto {\tilde{\nabla }}_{AA^{\prime }}{\tilde{\mu }}_{B^{\prime }}=\Omega^{w^{\prime }}(w^{\prime }\Upsilon_{AA^{\prime }}\mu_{B^{\prime }}+\nabla_{AA^{\prime }}\mu_{B^{\prime }}-\Upsilon_{B^{\prime }A}\mu_{A^{\prime }}), \end{array}\}$$

with related expressions for quantities with upper indices and/or with several such indices of either kind.

The equation (3.3) encounters difficulties in conformally curved spacetime if *s *> 1, as we find that there are unwanted consistency conditions. However, in the particular case of *gravity*, where *s *= 2, we find that these consistency conditions are automatically satisfied in vacuum. Yet, we do find a curious feature with regard to conformal invariance, as we shall be seeing shortly.

It is important, in what follows, to consider how null geodesics and their affine parameters behave under conformal rescaling. A *geodesic* is a curve *λ* that has a tangent vector *l*^*a*^ along it, for which we can take

3.8*a* $$l^{a}\nabla_{a}l^{b}=0,$$

and an *affine* parameter *r* for *λ*, associated with *l*^*a*^, is then a parameter *r* that satisfies

3.8*b* $$l^{a}\nabla_{a}r=1.$$

Taking $l^{a}=o^{A}o^{A^{\prime }}$, as in (2.14), we shall also require the flag plane of *o*^*A*^ (as depicted in figure 1, and see [21]), to be parallel propagated along *λ*, which we express as

3.8*c* $$l^{a}\nabla_{a}o^{B}=0,$$

along *λ*, this (and also (3.8*b*)) being conformally invariant, by (3.8).

We take note of the vacuum Bianchi identities, which when written in spinor form, using (2.16), take the form

3.9 $$\nabla^{AA^{\prime }}\Psi_{ABCD}=0,$$

(see [21,25]), this having the same form as (3.3). However, when we take note of the fact that the Weyl tensor *C_abcd_* is actually the *conformal curvature,* having the very specific conformal weight –2, we find that Ψ*_ABCD_* necessarily has weight 0, rather than the –1 required for the conformal invariance of (3.8). This leads us to consider that the spin-2 gravitational radiation field is really described by a symmetrical spinor quantity *ψ_ABCD_* which, though *equal* to the Weyl curvature spinor quantity Ψ*_ABCD_* when referred to the physical metric *g_ab_*,

3.10 $$\psi_{ABCD}=\Psi_{ABCD}$$

has conformal weight –1, so under conformal rescaling (3.1), (3.2),

3.11 $$\psi_{ABCD}\mapsto {\tilde{\psi }}_{ABCD}=\Omega^{-1}\psi_{ABCD}=\Omega^{-1}\Psi_{ABCD}=\Omega^{-1}{\tilde{\Psi }}_{ABCD}.$$

**Figure 3.**
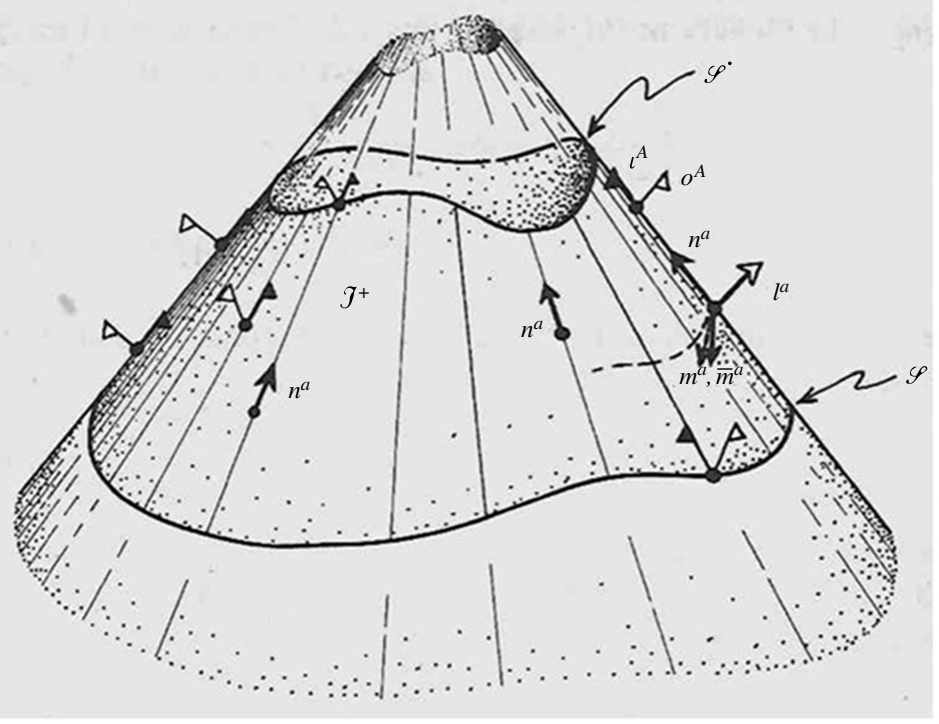
A picture of $I^{+}$. White flags represent *o*^*A*^ and black flags, $\iota^{A}$. Two cuts S and $S^{\prime }$ are indicated.

The significance, for us, of the quantity *ψ_ABCD_*, as opposed to Ψ*_ABCD_*, is that its conformal weighting, being that of a massless free field, allows it to retain a *finite* value at *conformal infinity* $I^{+}$, a concept that we now turn to. An impression of this notion of $I^{+}$ is provided by figure 3*.* The future bounding hypersurface $I^{+}$ is three-dimensional (though depicted in figure 3 as two-dimensional, with one spacial direction suppressed), providing a conformally smooth future-null boundary to the spacetime M, when it is itself regarded as a conformal manifold.

Although this procedure has been found to be very broadly applicable for Bondi-Sachs-type spacetimes, questions are sometimes raised about the generality of this procedure. There are, for example, situations that one could consider as ‘physically realistic' (such as those involving a pair of slowly in-spiraling bodies) whose outgoing gravitational waves could affect the smoothness of the conformal geometry at $I^{+}$. Moreover, in any case, one can certainly envisage all kinds of artificially introduced incoming radiation that could spoil the smooth geometry in the neighbourhood of $I^{+}$. I am adopting the viewpoint here that all such possibilities are really irrelevant to the utility of the concept of $I^{+}$, and that its assumed smoothness does not restrict our understanding of general gravitationally radiating systems involving their production of outgoing radiation. Moreover, at first sight one might worry that (3.11) could imply problems for ${\tilde{\psi }}_{ABCD}$ remaining finite at $I^{+}$, where Ω = 0. However, there is a theorem (see [28]), indicating that ${\tilde{\Psi }}_{ABCD}$ should vanish appropriately at $I^{+}$ in general situations describing outgoing gravitational radiation, thereby providing a finite ${\tilde{\psi }}_{ABCD}$ at $I^{+}$.

Figure 3 indeed gives us an impression of the kind of situation under consideration here. It depicts conformal infinity $I^{+}$, which represents the totality of limiting points (*r* → ∞) along the outgoing null geodesic generators of the *u *= const. coordinate hypersurfaces. See (1.1). The little white flags represent the limiting location of *o*^*A*^, using the description depicted in figure 1, with flagpole pointing in the direction of such null geodesic. We can locally choose a spin-frame {${\tilde{o}}^{A}$, ${\tilde{\iota }}^{A}$} at each point of $I^{+}$and because of (3.8*c*), we can demand that ${\tilde{o}}^{A}=o^{A}$, this being consistent with the parallel propagation of *o*^*A*^ along *λ* given by (3.8*c*). The choice of ${\tilde{\iota }}^{A}$ is not determined in this way, but we demand that its flagpole direction (i.e. that of *n^*a*^*) lies along the direction of the generator of the null hypersurface $I^{+}$ at that point.

Figure 3 illustrates this situation, where the little white flags represent the locations of the *o*^*A*^ spinors at $I^{+}$, and the little black flags the corresponding locations of the $\iota^{A}$ spinors. The roughly horizontal wavy cross-sections, or *cuts*, of $I^{+}$, which are illustrated in figure 3, may be considered as places where the coordinate *u* takes a particular value along the cut, and the white flags' flagpoles point out orthogonally along such a cut.

It should be made clear that the structure of $I^{+}$ itself is not dependent on any particular choice of family of such cuts. Any choice of cuts would be associated with a choice of *o*^*A*^ spinors (i.e. of *l*^*a*^ directions) there, since the spacelike two surfaces that are tangent to the cuts of $I^{+}$ there are defined as being orthogonal to the *l*^*a*^ directions there (being the 2-planes spanned by the real and imaginary parts of *m*^*a*^ there). In the Bondi-Sachs analysis, a particular restriction is made on the *u* coordinate that makes the family of cuts ‘parallel' to one another in a certain sense, although this restriction will not be of particular importance to us in what follows.

Nevertheless, it is worthwhile to point out the particular quantity *τ* that is made to vanish for the cuts to be ‘parallel' in the Bondi-Sachs analysis, as *τ* is one of the four *spin coefficients ρ*, *σ*, *κ*, *τ* [21,29], that are especially relevant to our considerations here, these being defined by

3.12 $$\kappa =o^{A}\nabla_{00^{\prime }}o_{A},\;\rho =o^{A}\nabla_{10^{\prime }}o_{A},\;\sigma =o^{A}\nabla_{01^{\prime }}o_{A},\;\tau =o^{A}\nabla_{11^{\prime }}o_{A}.$$

The quantity *κ* is a measure of the *curvature* of null curves *λ* lying along the flagpole directions of *o*^*A*^, so that the condition *κ* = 0 asserts the geodesic nature of these *λ* curves, see (3.8*c*), with the flag planes being taken parallel-propagated along *λ*. The modulus |*κ*| of the complex quantity *κ* provides a measure of the curvature of *λ* and arg*κ* tells us the direction of this curvature in relation to *o*^*A*^'s flag plane. The complex quantity *ρ* has a *real* part that describes the *convergence* of the family of *λ*-curves, the imaginary part describing a *rotation* of the flag planes along the *λ* directions (Im*ρ* being taken to be zero here). The complex quantity *σ* will be of particular importance to us later. It describes the *shear* of the family of *λ*-curves, the modulus of this complex quantity *σ* describing the magnitude of this shearing and the argument (phase) describing the direction away from the *λ*-curve of direction out from *λ* of maximum shearing. The quantity *τ* describes how *o*^*A*^ varies in a direction away from the null 3-plane element determined by *l*^*a*^, so that for the ‘parallel' cuts of the Bondi-Sachs system, we indeed demand *τ* = 0 all along $I^{+}$.

We now turn to the Bondi-Sachs definition of the total *mass* of a system, as defined at the ‘retarded time' defined by a particular cut C of $I^{+}$. We must bear in mind that the notion of ‘mass' of relevance here is to be what we should regard as a ‘time-component' of some notion of a ‘4-vector' of some appropriate kind. For this, we need the notion of the *future celestial sphere* $S^{+}$, of M, where each point of $S^{+}$ represents a *generator* of the null 3-cylinder $I^{+}$, these generators lying along the $\iota^{A}$ flagpole (i.e. *n*^*a*^) directions, being defined by fixed values of *θ* and *ϕ* in the original metric given in (1.2). It is helpful to re-express these coordinates in terms of the single complex variable

3.12*a* $$\zeta ={\text{e}}^{i\phi }\cot\frac{\theta }{2}.$$

So that the non-reflective conformal motions of (the *Riemann sphere*) $S^{+}$ are defined by bilinear transformations of *ζ*:

3.13 $$\zeta \mapsto \frac{\alpha \zeta +\beta }{\gamma \zeta +\delta },\;\text{where}\;\alpha ,\beta ,\gamma \;\text{and}\;\delta \;\text{are complex constants}.$$

(See e.g. [30].) The group defined by (3.13), being the non-reflective Lorentz group, leaves the general form of the Bondi-Sachs metric unchanged. We can regard the sphere $S^{+}$ as being the space of generators of $I^{+}$, each cut of $I^{+}$ providing a particular realization of $S^{+}$. Accordingly, we may also regard the transformations (3.13) as acting on any particular cut C itself. The notion of the direction of a *timelike vector*, in relation to C, would refer to the assignment of a particular unit sphere metric to $S^{+}$, consistent with its conformal structure. This would apply, in particular, to an energy-momentum vector.

In order to appreciate the Bondi-Sachs definition of energy-momentum, we need to consider the (limiting) *Weyl curvature* of M at C*.* For this, we need the values of the finite quantity ${\tilde{\psi }}_{ABCD}$, defined in (3.11), most particularly

3.14 $$\psi_{4}={\tilde{\psi }}_{1111},\;\psi_{3}={\tilde{\psi }}_{1110},\;\text{ and }\;\psi_{2}={\tilde{\psi }}_{1100}.$$

We also need the *shear σ* of the outgoing rays, as defined in (3.12) and the important, but not quite locally defined *news function N*, which can be expressed, equivalently, either as a time-integral of *ψ*_4_*,* or as an angular integral of *ψ*_3_ around the cut, where we get the same answer either way even though the value of *N* at a point *p* is not completely determined in terms of quantities defined locally at *p* (see [31], p. 427]).

The Bondi-Sachs definition of the *mass M* at the cut C is then given by an integral over the cut of the quantity

3.15 $$\psi_{2}-\sigma N,$$

(with the appropriate numerical factors), and the Bondi-Sachs mass-loss formula asserts that if the integral of (3.14) is performed over a *later* cut C^′^ (i.e. ‘higher up', in figure 3), then this later value is reduced from the earlier value by (4πG)^–1^ times the integral of |*N*|^2^ taken over the region of $I^{+}$ between these two cuts, with an appropriate additional contribution from the energy-momentum tensor of any ordinary matter fields that might also be present (see [22, vol. 2, p. 426]).

## 4. Ezra T. Newman's approach to equations of motion

The Bondi-Sachs mass-loss result was a clear landmark in understanding the contribution from the gravitational field in GR to the mass-energy of a gravitating system. Much of the early work in this area (though *not* the key formula (3.15)) depended upon coordinate systems that respect the ‘parallel' (*τ * = 0) asymptotic nature of the *u *= const. coordinate hypersurfaces. The general study of such systems led to what is referred to as the BMS (Bondi-Metzner-Sachs) group [11,32]; see also [33]. However, it is not at all clear what physical role the *τ *= 0 condition actually plays, since it is not necessary for the mass-loss result, and a physical role for the BMS group does not seem to be really compelling. Indeed, there is another approach to restricting the large coordinate freedom, for such spacetimes, which has led to some very different and highly intriguing implications. This is the H*-space* approach due to Ezra T. Newman [34,35].

The idea of H-space is to drop the ‘parallel' (*τ *= 0) character of the *u *= const. coordinate hypersurfaces (i.e. of the family of cuts of $I^{+}$) and replace it with a requirement that the asymptotic *shear σ* of these hypersurfaces must vanish. At first sight, this would appear to be far too strong a requirement, since *σ* is a *complex* quantity, whereas the freedom per point of a cut is just *one real* parameter, this freedom being simply that of sliding up or down the intersection of the cut with each individual generator of $I^{+}$. This freedom falls far short of what would be needed for what Newman would refer to as a ‘good cut’, for which *σ* would have to vanish all over the cut. Nevertheless, Newman was not to be daunted by such a blatant fact, allowing his cuts to be *complex,* so that each of its points would be allowed to meet the generators of $I^{+}$ in *complex* points, not necessarily real ones!

This raises a number of technical issues. We are now concerned with the *complexification* $CI^{+}$, of $I^{+}$, rather than with $I^{+}$ itself. This, in turn, requires that M be an *analytic* manifold, with an analytic metric. This, in itself, is not a troublesome restriction, so long as we are not considering situations involving impulsive waves, or the like, since we can always approximate a reasonably smooth metric by an analytic one. Problems do not arise with Newman's construction provided that we need not venture too far from the real spacetime under consideration, so we can take M to be analytic, with an analytic metric so that a complexification CM exists, which provides an open neighbourhood of M, which need not extend significantly far away from M itself.

A more serious issue is that the ‘complexification' of a quantity that is already complex, such as *σ*, is not so straightforward. In effect, we have to consider two *distinct* complex quantities *σ* and $\tilde{\sigma }$, where for the real spacetime M, $\tilde{\sigma }=\bar{\sigma }$ (the complex conjugate of *σ*), whereas for CM, the complex quantities *σ* and $\tilde{\sigma }$ become *independent* of one another. Thus, in general, we cannot expect that our notion of ‘good cut' involves making *both σ* and $\tilde{\sigma }$ vanish at once. Instead, we need to make a choice as to which *one* of *σ* or $\tilde{\sigma }$ is to vanish (where the other is not required to vanish) in order to provide us with the *good cuts,* the family of which is to provide us with the definition of H-space, the other choice giving us the complex conjugate $\bar{H}$*-*space.

It turns out that H*-*space is indeed a 4-complex-dimensional manifold [34] and which, somewhat remarkably, turns out to have a complex metric that automatically satisfies Einstein's vacuum equations (vanishing complex Ricci tensor) [35,36]. Moreover, this construction had an important influence on twistor theory [35], leading to what is referred to as the ‘nonlinear graviton construction' [36]. This, in effect, provides us with the general solution of the ‘anti-self-dual' Einstein vacuum equations (e.g. [37]; see also [38,39], and [40]), although these are necessarily *complex* solutions, or else real ones but with a different signature from the Lorentzian one needed for a physical spacetime. For gauge fields, see also [41], and [42].

An application of a very different kind is Newman's discovery that numerous aspects of equations of motion for small bodies in GR (touched upon in §1) can be much more readily obtained using H*-*space techniques than by the standard procedures [43,44]. This development is technically impressive, yet it perhaps lacks a convincing underlying justification for deriving equations of motion in this way. It would appear to be an important development for the theory of equations of motion to find such a justification.

A central ingredient of Newman's H*-*space approach to equations of motion dates back to his observation that the Kerr metric for a rotating black hole can be thought of as being, in a certain sense, a displacement of the Schwarzschild metric in a *complex* spatial direction. When applied to the electrically charged version of the Schwarzschild metric, namely the Reissner-Nordström metric, [45] and [46] one obtains the charged version of the Kerr metric, sometimes referred to as the Kerr-Newman metric [27]. In this sense, we can regard such a complex spatial displacement as corresponding to the acquirement of an intrinsic *angular momentum*, or ‘spin'. This same general idea, when applied to the world-line of a charged particle, provides it with a magnetic moment. These complex displacements lead us to an H*-*space description.

There is some good reason to expect that this could be related to another body of results, going back to 1965, but which have remained distinctly enigmatic ever since. These results refer to a family of quantities referred to as ‘NP constants' (Newman-Penrose constants) [21,22,30,47–49] but rarely accepted as genuine physical phenomena, since they give an impression of being at odds with accepted physical theory. In fact, this is not so, but these quantities do shed a distinctly unusual light on conventional physical expectations, seeming to suggest that certain physical quantities would have to be conserved which are certainly *not* conserved, thereby evoking negative reactions from some distinguished theoreticians, ranging from serious discouragement [50] to actual disbelief [51,52].

To be more explicit, for a s*tationary* asymptotically flat spacetime, the gravitational NP constants can be identified as the combination of gravitational quantities given by:

4.1 $$(\text{mass})\times (\text{complex quadrupole moment})-{\left(\text{complex dipole moment}\right)}^{2},$$

where the *complex* dipole moment has an imaginary part that is the angular moment and there is a corresponding imaginary contribution to the quadruple moment (see [22], p. 428] and compare with~[53]). In view of such an explicit expression as (4.1), the above skeptical reactions are perhaps not surprising. For example, the constancy of the NP quantities for pure gravity implies that a stationary system, say with a non-zero quadrupole moment, but with no dipole moment or angular momentum cannot, by the emission of gravitational waves, evolve, after a finite period of time, to the same situation except with a changed quadrupole moment! The seeming contradiction with the descriptions given in §1, that gravitational waves can carry away quadrupole moment while leaving the dipole and angular momentum unchanged, is nevertheless resolved by the fact that the presence of back-scattered gravitational radiation would provide an additional contribution that would (surprisingly) prevent the state ever becoming sufficiently stationary for any contradiction with NP constancy to arise! (See [49] for a more detailed discussion of this situation.) Nevertheless, this conclusion is very non-intuitive, and I am proposing, later in this article, that a completely different light on the underlying significance of the conservation of NP quantities can be provided which could well relate to Newman's remarkable procedure for deriving equations of motion.

For this, we need to re-examine what was my own initial route to anticipating an NP constancy—which seems to have been somewhat complementary to the route taken by Newman, his route perhaps having had a more direct connection with the multipole issue, which I had not anticipated. I had been concerned with a quite different question, namely the initial value problem, in Minkowski space M, for the massless free-field equation (3.3)

4.2 $$\nabla^{AA^{\prime }}\phi_{AB\ldots E}=0,$$

where the field *ϕ_AB_*_…_*_E_* is given on an initial *null* hypersurface N. We wish to determine *ϕ_AB_*_…_*_E_* at some arbitrary point *P*, lying in the region to the future of N, by performing an integral of an appropriate quantity defined by *ϕ_AB_*_…_*_E_* on N. It turns out that this can be achieved by performing an integral over the two-dimensional intersection

**Figure 4.**
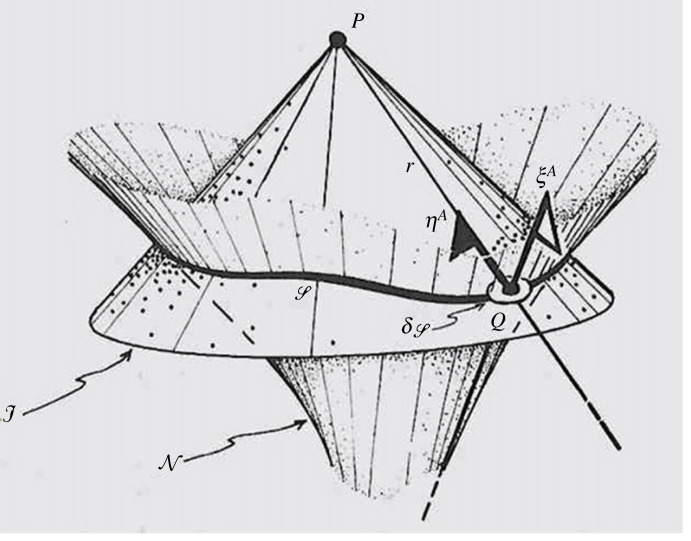
Geometry for the GKd solution of the initial value problem for a massless free field.

4.3 S=N∩J,

of N with the (past) light cone J of *P*. At each point of S, we choose a spin-frame {*o*^*A*^,$\iota^{A}$} where the flagpole of *o*^*A*^ points along the null generator of N and that of $\iota^{A}$, along the corresponding generator of J, at the same point of S, represented as white and black flags, respectively, at a typical point *Q* of S (figure 4) for the geometry involved (with white and black flags respectively, as in figure 3). The particular scalar quantity to be integrated over S, together with its surface area 2-form dS, is defined in terms of the operator *ϱ*_0_, as given by

4.4 $$\varrho_{0}\phi_{0}:=(\nabla_{00^{\prime }}-(n+1)\rho )\phi_{00\ldots 0},$$

where *n* is the number of indices of *ϕ_AB_*_…_*_E_* (i.e. twice the spin). Our integral to provide us with the field at *P* is then simply

4.5 $$\phi_{AB\ldots E}(P)=\frac{1}{2\pi }\oint_{S}\iota_{A}\iota_{B}\ldots \iota_{E}\frac{\varrho_{0}\phi_{0}}{r}\text{d}S.$$

The value of *r* is the affine distance of *Q* to *P* where *Q* is the point at which *ϕ_AB_*_…_*_E_* is being examined in (4.4 and (4.5), and *r* is the affine distance *QP* scaled in terms of *n*^*a*^ = $\iota^{A}\iota^{A^{\prime }}$, so that we can write

4.6 QP=r n

(figure 4). It might be thought that there is a topological issue of defining the directions of the flag-planes of *o*^*A*^ and $\iota^{A}$ consistently over the whole of S, but this is not really a problem, because the integrand in (4.5) is insensitive to the phase involved in the choice of spin-frame. I refer to the expression (4.5) as the *generalized* *Kirchhoff–d’ Adhémar formula* [54,55] or the GKd formula, whose early work I was able to generalize to arbitrary spin in around 1967 [56–58].

In fact, the expression (4.5) works just as well in conformally flat spacetime (see [21] p. 395), using the appropriate conformally invariant expression for the operator *ϱ* of (4.4), which acts in the direction of *o*^*A*^'s flagpole direction at the point *Q* in figure 3. The integral is to be performed at the point *P*, the spin frame {*o*^*A*^, $\iota^{A}$} being taken to be parallel-propagated along each generator of J from *Q* to *P*. This conformal invariance allows us to regard the null cone J to be the future conformal infinity of an asymptotically flat spacetime M

4.7 $$J=I^{+},$$

and then S becomes a particular *cut* of $I^{+}$.

Now, one way of proving the GKd formula is to examine how the integrand of (4.5) might vary as the cut S of t J might vary as it is moved up the generators of J, it being found that the integral in (4.5) actually remains constant as the cut is moved up towards *P*, so that all that remains is to show that the limiting value is indeed the required value of *ϕ_AB_*_…_*_E_* at *P*. Such a procedure fails, in general, if the ambient spacetime manifold is conformally curved, because of troublesome curvature terms that do not vanish in general. However, what is remarkable is that if we choose J to be $I^{+}$ as suggested above, then all these troublesome terms actually *vanish*, so that our integral does indeed provide a quantity that is absolutely conserved without the need of a contribution such as the of |*N*|^2^ term in the Bondi-Sachs theory (referred to at the end of §3) for the loss of total mass by gravitational radiation. Instead, we now find these curiously *absolutely* conserved quantities, referred to as ‘NP constants', which seem to have no *physical* basis for their conservation, according to conventional theory!

For a spin *s* massless field, the number of independent such complex quantities would be the number of complex components of *ϕ_AB_*_…_*_E_*, with 2 *s* symmetrical 2-spinor indices, namely

4.8 2s+1,

complex numbers, which provide us with 10 real numbers for gravity and 6 for electromagnetism, the two known massless fields in physics. It is striking that, moreover, in the Einstein–Maxwell theory, we retain the six quantities for the source-free electromagnetic field, but the situation is changed for the gravitational field, since the Maxwell field itself provides a source for the gravitational field. Nevertheless, we can maintain the exact conservation of 10 gravitational quantities by including a contribution involving the electromagnetic field [59].

In view of all these remarkable mathematical facts, it is very hard to believe that these exactly constant quantities cannot have some kind of basic physical role to play in our actual physical world. I wish to put forward a different perspective on the quantities from previously, which might, indeed, provide such ‘NP constants' with a quite different kind of physical rationale for their constancy. To explain what I have in mind, let me first return to the interpretion of these constants given above, addressing a point not raised before in this article. First of all, where do we *locate* the actual ‘spinor' *ϕ_ABCD_* that encapsulate the 10 NP constants in the gravitational case? If we think of the conformal picture of Minkowski space M, our point *P* where this *ϕ_ABCD_* would be located would be the ideal point i^+^, representing future timelike infinity for M (see [49]). For an asymptotically flat M, we might still get such a point i^+^, if all the material in that model evaporates away in radiation, but in most cases, we might consider that some material remains, so that our future timelike infinity point ‘i^+^’ would be likely to be *singular* (and perhaps even a singular spacelike surface). Nevertheless, our NP ‘GKd' integral would provide us with the value *ψ_ABCD_*, basically the *conformal curvature tensor*, at this nonexistent ‘virtual point' i^+^. We appear to have to think of *ψ_ABCD_* as providing a kind of ‘background’ that cannot be altered by whatever the system might do in its later activity.

**Figure 5.**
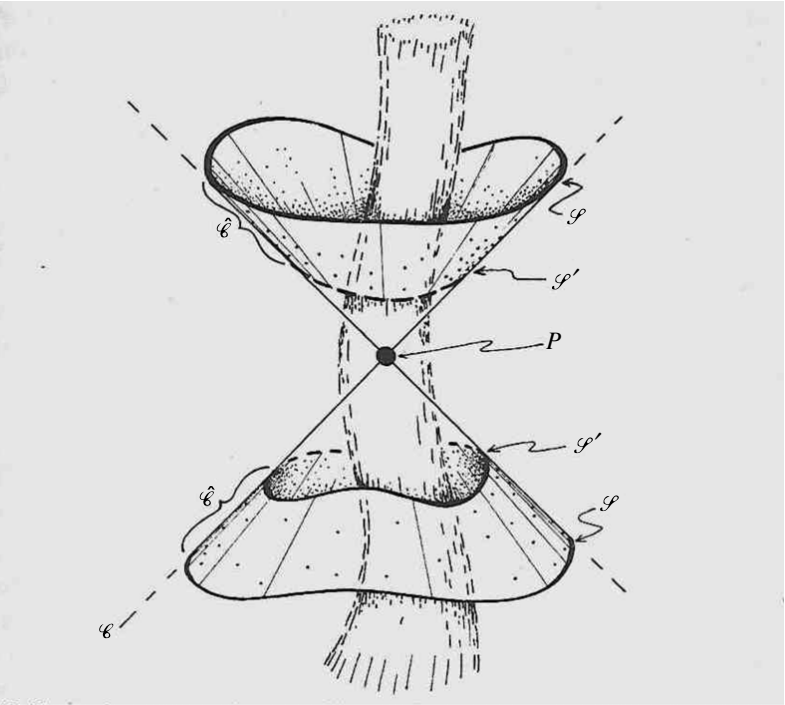
The G*Kd* integrals surrounding the central body eliminate the field of the body to give the background field at *p*.

It seems to me that this all makes much more sense if we think of the NP constants in a quite different context, namely that of *equations of motion*. In that subject, we are thinking of taking a quite different kind of limit where, rather than looking at a large-scale limit of greater and greater distances away, we are more concerned with small-scale limits of tinier and tinier distances. As already mentioned in §1, equations of motion have to deal with taking an inward limit and trying to ascertain what the ‘background field' that a tiny particle might be responding to. Again, it is a question of seeking a ‘background field', but on a small, rather than a large scale. The idea is to think of the NP constants as not really applying to ‘infinity' but to what would ‘look like' infinity from the very small scale that we are concerned with. Thus the ‘$I^{+}$’ that we are now concerned with could be thought of a fairly small distance away from the particle whose motion we are examining, but which looks more and more like the conformal infinity the closer to the particle we get. See figure 5 for a picture of what I have in mind. The scale of the picture is supposed to be small enough that we can ignore spacetime curvature's nonlinear effects, so that we can regard the GKd integral to be valid, at least in regions that are not too close to the particle that we are concerned with.

In figure 5, the vertical tube in the middle represents the history of such a small body, whose motion we wish to calculate, but we are concerned to eliminate the field of the body itself. We consider the point *P* at the centre of the picture, lying within that body. There are four (irregular-looking) boundary loops in the picture (actually of spherical topology), and we might perform a GKd integral over any one of the four. Each would provide a version of the background field of the body (since the contribution from the body's field would not contribute, by arguments effectively provided above), but with now the point *P* replacing the i^­+^ of our earlier discussion. Accordingly, the upper two integrals would give the same ‘retarded' answer and the lower two, the same ‘advanced' answer. Very possibly *all* are the same because all are finite, so no ‘renormalization’ is required. All this requires further study.

Finally, there is a probable connection with Newman's work on his H*-*space approach. It seems to me that this is very likely, since both are deeply connected with twistor theory. The H*-*space connection has already been mentioned and the GKd integral is almost equivalent to the basic twistor contour integral for massless free fields [57,58].

Thanks go to Jörg Frauendiener for help with references and to John Moussouris for financial assistance.

This paper is dedicated to the memory of Ezra (Ted) Newman.

## Data Availability

This article has no additional data.
